# Lipid transfer protein syndrome in a Northern European patient: An unusual case report

**DOI:** 10.3389/fmed.2023.1049477

**Published:** 2023-02-07

**Authors:** E. Albert, T. Walsemann, J. Behrends, U. Jappe

**Affiliations:** ^1^Division of Clinical and Molecular Allergology, Research Center Borstel, Leibniz Lung Center, Airway Research Center North (ARCN), Member of the German Center for Lung Research, Borstel, Germany; ^2^Core Facility Fluorescence Cytometry, Research Center Borstel, Leibniz Lung Center, Borstel, Germany; ^3^Interdisciplinary Allergy Outpatient Clinic, Medical Clinic III - Pneumology, University of Lübeck, Lübeck, Germany

**Keywords:** non-specific lipid transfer protein, LTP, LTP syndrome, food allergy, basophil activation test, exercise-induced anaphylaxis

## Abstract

Non-specific lipid transfer proteins (nsLTPs) as the primary sensitizer in plant-food allergic patients used to be seen primarily in the Mediterranean area. However, more recently, increasing numbers of clinically relevant sensitizations are being observed in Northern Europe. We herein report an unusual case of a woman who developed an anaphylactic reaction during a meal including a variety of different foods ranging from fruits and nuts to oats, wheat, and salmon. Allergy diagnostics showed no Bet v 1 sensitization but an nsLTP-mediated food allergy. Despite the much more prominent birch food syndrome in Central and Northern Europe, LTPs should be considered disease-causing agents, especially for patients developing severe reactions after consuming LTP-containing foods.

##  Introduction

Non-specific lipid transfer proteins (nsLTPs) are small, highly structurally stable proteins found in various plant foods and pollen. Their structure contains four disulfide bonds that are responsible for their resistance to thermal processing as well as gastrointestinal digestion (1). These characteristics provide them with the ability to induce primary gastrointestinal sensitization. LTPs as a major cause of food allergy have so far been mainly recognized in the Mediterranean area, while reports of LTP-mediated food allergy in Northern Europe have been rare (2). In this area, the birch food syndrome is the dominant cause of allergic reactions to various plant foods, whereas LTPs as the cause of pollen-associated or primary food allergy still seem to be rare (2). Patients with birch food syndrome sometimes simultaneously present with co-sensitization to LTPs like Pru p 3 (peach), Cor a 8 (hazelnut), Mal d 3 (apple), and others, but these cases mostly show mild allergic reactions (3). In contrast to that, LTP-mediated food allergy often presents with much more severe manifestations (4). Patients with LTP syndrome experience reactions to multiple plant foods due to the wide distribution of these allergens in plant sources (5). The severity of reactions additionally increases with the number of LTP sensitizations (2). In some cases, the manifestation of symptoms in allergic patients can depend on the presence of a cofactor, such as exercise (6). These cofactors potentially amplify allergic

reactions by decreasing the amount of allergen needed to induce reactions in patients with lower allergen sensitization (7). Currently, the understanding of the pathomechanism behind this phenomenon remains to be fully understood. One proposed mechanism behind exercise-induced anaphylaxis involves changes in the mucosal permeability resulting in increased allergen exposure (7). We herein describe the case of a woman who developed two separate allergic reactions after consumption of plant foods, the first one presenting as anaphylaxis and the second one as an oral allergy syndrome. In the case of the anaphylactic reaction, the cofactor exercise was present before consumption. Allergy diagnostics revealed evidence of an LTP-mediated food allergy.

## Case description

**FIGURE 1:**
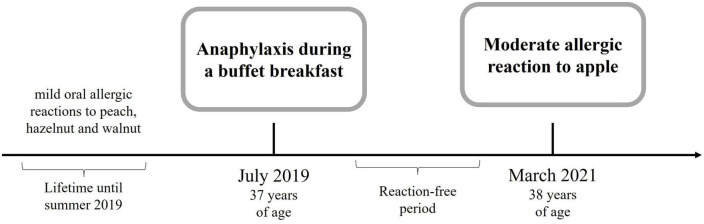
Timeline of the patient’s allergological history.

A 37-year-old woman presented to the emergency department with an anaphylactic shock including burning and tingling of her tongue, emesis and diarrhea, generalized urticaria, facial angioedema progressing to dizziness, and difficulties in swallowing and breathing due to swelling of her throat. Her past medical history included lactose intolerance and sensitization to house dust mites, and she also experienced non-allergy related diseases including Hashimoto’s thyroiditis and autoimmune uveitis. The patient neither suffered from allergic rhinitis, asthma, or atopic eczema nor did she take any medication regularly. This anaphylactic reaction happened during a buffet breakfast with a multitude of allergen sources including salmon, pine nuts, walnuts, sesame, wheat, oats, mustard, honey, orange juice, melon, apple, grapes, grapefruit, blood orange, and coffee. Thorough clinical history revealed that the patient underwent physical exercise 60 min before her breakfast. She remained incident-free for more than one and a half years until she presented a second time to a primary care physician with an intense tingling of the tongue after consumption of an apple crumble and coffee (Figure 1). No other LTP-containing foods were consumed in parallel. Almost all the ingredients of the apple crumble she had been eating daily, besides the apple itself, which is why the apple was considered suspicious of being the allergy trigger. This time physical activity as a cofactor was excluded; however, menses and the intake of non-steroidal antiphlogistics are probable. Medical history further revealed that the patient had experienced itchy eyes, an erythematous itchy reaction at her neck, and swelling of her lips after the consumption of peach many years ago, which was her very first allergic reaction. The consumption of walnut and hazelnut was accompanied by oral allergy syndrome, and in the case of hazelnut (hazelnut flour in a bread roll), the eyes were itchy and the lips were swollen in addition to oral allergy syndrome.

### Diagnostic assessment

**FIGURE 2:**
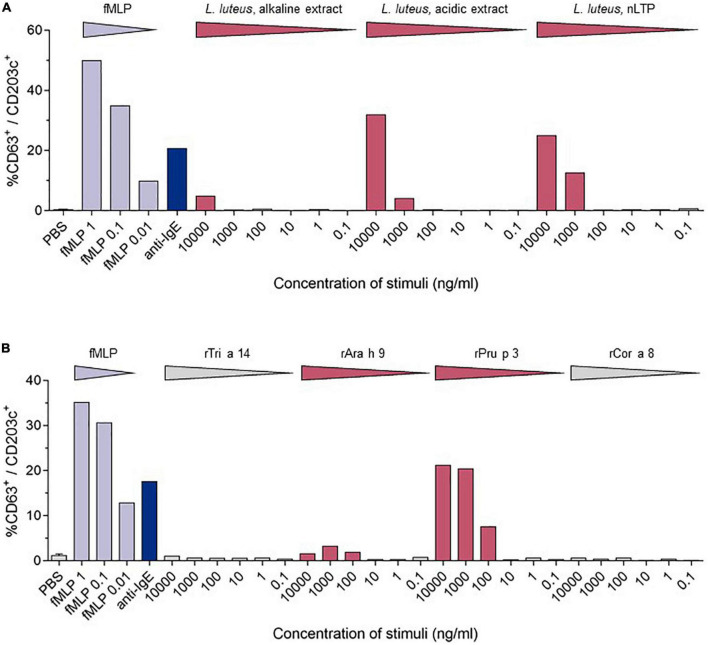
Comparison of the stimulation-induced basophil activation levels. Basophil activation was measured by the percentages of CD63 positive basophils (CD203c^+^/FcϵRIα^+^). PBS—phosphate-buffered saline, fmIP- formyl-, methionyl-leucyl phenylalanine. **(A)** Results of the BAT for LTPs from *Lupinus luteus* [acidic extract; alkaline extract; nLup l 3 (LTP)] (10). **(B)** BAT results from wheat (rTri a 14), peanut (rAra h 9), peach (rPru p3), and hazelnut (rCor a 8).

Diagnostic assessment: skin prick testing revealed sensitization to peach (3 mm) and hazelnut (5 mm). Herring, hen’s egg extract, crab extract, peanut extract, walnut, almond, cow’s milk, soya milk, wheat flour, lupine flour, raw apple, raw carrot, raw celery, and raw tomato were negative. The positive control (histamine) reaction was 5 mm. The concentration of specific IgE-antibodies to different allergen extracts and single allergens was determined by Immuno Solid-phase Allergen Chip (ImmunoCAP; Phadia; Uppsala, Sweden). Total serum IgE was slightly elevated (110 kU/L; normal <100 kU/L). Specific IgE (sIgE) to allergen extracts was positive for pine nut, lupine seed, mugwort, olive tree, *Dermatophagoides farinae*, and *D. pteronyssinus*, and negative (<0.01 kU/L) for wheat and mustard. Specific IgE to single allergens was positive in descending concentration for the nsLTPs of peach (rPru p 3), apple (rMal d 3), mugwort (n Art v 3), walnut (rJug r 3), rAra h 9, wheat (rTri a 14), hazelnut (rCor a 8), and the olive tree (r Ole e 7). These results are shown in Table 1. The detailed medical history led to the suspicion of an LTP-mediated reaction to food. This suspicion was confirmed by *in vitro* allergy diagnostics showing no IgE reaction toward birch pollen [Bet v 1, Bet v 2 (profilin), and Bet v 4 (polcalcin)] but positive reactions to multiple lipid transfer proteins (Table 1). In addition to specific IgE detection, a basophil activation test (BAT) with the lipid transfer proteins from lupine (*Lupinus luteus*, originates from the research group Prof. Jappe) (Figure 2A) and from wheat, peanut, peach, and hazelnut (Indoor Biotechnologies Ltd., Cardiff, UK) (Figure 2B) was performed, which revealed a positive result for peach, lupine, and peanut in descending order and was negative for wheat and hazelnut. The LTPs from apple and pollen were not included as they were not available for BAT. There were no IgE reactions in ImmunoCAP to the peanut storage proteins Ara h 1–3 and Ara h 8, the Bet v 1-homolog in peanut, so the reaction to lupine LTP is most probably a cross-reactivity between LTPs. With sIgE of five or more LTPs, reactions are usually severe, leading to the diagnosis of LTP syndrome in this patient. The patient was advised to avoid the consumption of apples in any form and was equipped with an emergency set consisting of an adrenaline auto-injector, glucocorticosteroids, and antihistamines. There has been no re-presentation since.

**TABLE 1:** *In vitro* allergy diagnostics: Specific IgE-antibody detection results (ImmunoCAP).

Allergen source	Protein family/ biochemical name	Allergen component	IgE (kU/L)	CAP class
Peach (*Prunus persica*)	**nsLTP**	**rPru p 3**	8.7	3
Peach (*Prunus persica*)	Bet v 1-homolog	rPru p 1	0.13	0
Peach (*Prunus persica*)	Profilin	rPru p 4	<0.01	0
Peach (*Prunus persica*)	Gibberellin-regulated protein	rPru p 7	<0.01	0
Apple (*Malus domestica*)		Extract	4.5	3
Apple (*Malus domestica*)	nsLTP	**rMal d 3**	7.97	3
Apple (*Malus domestica*)	Bet v 1-homolog	rMal d 1	<0.01	0
Walnut (*Juglans regia*)		Extract	3.22	2
Walnut (*Juglans regia*)	nsLTP	**rJug r 3**	3.22	2
Walnut (*Juglans regia*)	2S albumin	rJug r 1	<0.01	0
Lupine seed (*Lupinus albus*)		Lupine seed extract	2.3	2
Wheat (*Triticum aestivum*)	nsLTP	**rTri a 14**	1.63	2
Wheat (*Tr. aestivum*)	Omega-5 gliadin	rTri a 19	<0.01	0
Wheat (*Triticum aestivum*)		Wheat extract	<0.01	0
Pine nut		Pine nut extract	1.25	2
Hazelnut (*Corylus avellana*)	nsLTP	**rCor a 8**	0.66	1
Hazelnut (*C. avellana*)	11S seed storage globulin	rCor a 9	<0.01	0
Hazelnut (*C. avellana*)	2S albumin	rCor a 14	<0.01	0
Peanut (*Arachis hypogaea*)	nsLTP	**rAra h 9**	3.72	2
Peanut (*A. hypogaea*)		Extract	1.21	1
Peanut (*Arachis hypogaea*)	7S globulin	rAra h 1	<0.01	0
Peanut (*Arachis hypogaea*)	2S albumin	rAra h 2	<0.01	0
Peanut (*Arachis hypogaea*)	11S globulin	rAra h 3	<0.01	0
Peanut (*Arachis hypogaea*)	Bet v 1-homolog	rAra h 8	<0.01	0
Mustard		Mustard extract	<0.01	0
Mugwort		Mugwort extract	2.08	2
Mugwort	nsLTP	**nArt v 3**	4.08	3
Olive tree (*Olea europaea*)		Olive tree extract	0.18	0
Olive tree	nsLTP	**rOle e 7**	0.69	1
Pellitory (*P. juglans*)		Extract	<0.01	0
*Parietaria juglans*	nsLTP	**rPar j 2**	<0.01	0
Birch pollen (*Betula verrucosa*)	PR 10-Protein	rBet v 1	0.17	0
Birch pollen (*Betula verrucosa*)	Profilin	rBet v 2	<0.01	0
Birch pollen (*Betula verrucosa*)	Polcalcin	rBet v 4	<0.01	0
Timothy (*Phleum pratense*)	Polcalcin	rPhl p 7	<0.01	0
Timothy (*Phleum pratense*)	Profilin	rPhl p 12	<0.01	0

## Discussion

Outside the Mediterranean area, LTP-mediated allergic reactions remain a rare diagnosis, and a full understanding of these geographical differences is still to be developed. Northern European countries have a higher load of birch pollen in comparison to Mediterranean countries. This high load of birch pollen with the dominance of Bet v 1 as a major allergen and primary sensitizer is suspected to decrease the probability of primary sensitizations to lipid transfer proteins in pollen, leading to mostly mild allergic reactions in LTP-sensitized Northern European patients (8). In contrast to that, LTP-mediated allergy can provoke various and more severe symptoms, including urticaria, nausea, diarrhea, angioedema, dizziness and even swelling of the throat, dyspnea, as well as cardiovascular disruptions. In our case, LTP-mediated anaphylaxis was induced by the consumption of multiple LTP-containing foods with preceding physical activity as a cofactor. The absence of typical birch pollen allergy symptoms like allergic rhinitis combined with the missing Bet v 1 sensitization led to the exclusion of the diagnosis of a birch pollen food syndrome. Our patient showed a strong sensitization to peach (rPru p 3), apple (rMal d 3), a moderate sensitization to walnut (rJug r 3) and wheat (rTri a 14) nsLTPs, as well as a low sensitization to the hazelnut nsLTP (rCor a 8). For peach, walnut, and hazelnut, the patient reported previous mild allergic reactions, which according to ImmunoCAP-results are not based on Bet v 1 or panallergens like profilins and polcalcins, but LTPs. The skin prick test and IgE-antibody assays can only detect sensitization and do not provide proof of allergy (9). The basophil activation test mimics the allergic reactions *in vitro*, and after optimization, discriminates between peanut-allergic and -sensitized individuals (9). For this patient, the BAT was performed using lupine, which is a legume like peanut, and the lipid transfer protein from yellow lupine seeds (nLup l 3), which was first identified and purified by Jappe et al. (10), and was, therefore, the original part of the “tool box” of the research group. After having obtained a positive reaction in BAT as proof-of-principle, additional commercially available LTPs (rTri a 14, rAra h 9, rPru p 3, and rCor a 8) were used in BAT. Although lupine seeds or lupine products were not knowingly a part of the buffet breakfast and the cross-reactive legume peanut is consumed daily without symptoms, this result together with the strong reaction to Pru p 3 and additional weaker reaction to Ara h 9 supports the diagnosis “LTP syndrome” in which patients are polysensitized to multiple LTPs from different food sources (1). During the anaphylactic event, the patient consumed multiple LTP-containing foods. None of them were identified as a new food source for the patient, which is why it is unlikely that anaphylaxis was induced by a single newly introduced allergen/allergen source. We propose it to be more likely that physical activity might have decreased the threshold of allergy induction. That, in combination with a high load of consumed LTP-containing foods, has potentially led to the severity of her reaction. This hypothesis of the LTP amount consumed as a risk factor in our patient is supported by the fact that the patient has not suffered a comparable allergic reaction before. The hypothesis is also supported by a recently published study on 67 Spanish patients with LTP-related anaphylaxis, 55/67 with anaphylaxis and a total of 134 anaphylactic reactions and 12/67 with anaphylactic shock and a total of 16 reactions (11). Another aspect in question is the route of sensitization. Possible routes include sensitization through cutaneous, gastrointestinal, and inhalant exposure (1). Asero reported peach-induced contact urticaria showing an association with nsLTPs (12). Primary gastrointestinal sensitization is a common cause of food allergy. For Pru p 3, Tordesillas et al. showed that it crosses the gastric barrier, which could be a reason for primary sensitization *via* the gastric route (13). Reactions to plant food sources can also occur *via* the inhalant route. One example is the birch-food syndrome describing cross-reactivity to vegetables and fruits containing Bet v 1-like proteins. Other inhalant allergen sources contain nsLTPs, which could lead to sensitization to nsLTPs *via* the inhalant route (14). Pollen nsLTPs like Ole e 7 from the olive tree, Art v 3 from mugwort, and Pla a 3 from the plane tree share partial cross-reactivity with *Rosaceae* nsLTPs and could be responsible for primary sensitization (15). As mugwort is especially common also in Northern Europe, we tested for the respective specific IgE-antibodies. The patient was indeed sensitized to the mugwort nsLTP nArt v 3. The patient originates from Northern Germany. There were no journeys to the Mediterranean area longer than 2 weeks. As the IgE concentrations to inhalant allergen sources and their LTPs were considerably lower than for peach and apple LTPs, we assume that the sensitization has most probably occurred *via* the ingestion of peach (see the first allergic reaction she has ever experienced.). This assumption is supported by the dominant sensitization to rPru p 3 in ImmunoCAP and the strong reaction to rPru p 3 in the BAT.

## Conclusion

An LTP-mediated allergy can provoke potentially life-threatening allergic reactions. Despite the much more prominent birch food syndrome in Central and Northern Europe, LTP allergens should be considered disease-causing agents and included in allergy diagnostic tests, especially for patients who experience severe reactions after consuming LTP-containing foods.

## Data availability statement

The original contributions presented in this study are included in this article/supplementary material, further inquiries can be directed to the corresponding author.

## Author contributions

EA wrote the manuscript and designed the Figure 1. TW planned, measured and analyzed BAT experiments and data, and designed the Figure 2. JB measured and analyzed BAT experiment and data. UJ diagnosed the patient, wrote and obtained ethical approval, wrote and revised the manuscript, and wrote the Table 1. All authors approved the final version of the manuscript.
